# Gene Expression-Genotype Analysis Implicates *GSDMA*, *GSDMB*, and *LRRC3C* as Contributors to Inflammatory Bowel Disease Susceptibility

**DOI:** 10.1155/2015/834805

**Published:** 2015-09-21

**Authors:** Jan Söderman, Linda Berglind, Sven Almer

**Affiliations:** ^1^Division of Medical Diagnostics, Ryhov County Hospital, 55185 Jönköping, Sweden; ^2^Department of Medicine, Solna, Karolinska Institutet, 17176 Stockholm, Sweden; ^3^Centre for Digestive Diseases, Karolinska University Hospital, 17176 Stockholm, Sweden

## Abstract

To investigate the biological foundation of the inflammatory bowel disease (IBD), ulcerative colitis and Crohn's disease, susceptibility locus rs2872507, we have investigated the expression of 13 genes using ileal and colonic biopsies from patients with IBD (inflamed and noninflamed mucosa) or from individuals without IBD (noninflamed mucosa). The susceptibility allele was consistently associated with reduced expression of *GSDMB* (*P* = 4.1 × 10^−3^–7.2 × 10^−10^). The susceptibility allele was also associated with the increased expression of *GSDMA* (*P* = 1.6 × 10^−4^) and *LRRC3C* (*P* = 7.8 × 10^−6^) in colon tissue from individuals without IBD and with the reduced expression of *PGAP3* (IBD; *P* = 2.0 × 10^−3^) and *ZPBP2* (Crohn's disease; *P* = 7.7 × 10^−4^) in noninflamed ileum. Inflammation resulted in the reduced colonic expression of *ERBB2*, *GRB7*, *MIEN1*, and *PGAP3* (*P* = 1.0 × 10^−4^–1.0 × 10^−9^) and the increased colonic expression of *IKZF3* and *CSF3* (*P* = 2.4 × 10^−7^–3.5 × 10^−8^). Based on our results and published findings on *GSDMA*, *GSDMB*, *LRRC3C*, and related proteins, we propose that this locus in part affects IBD susceptibility via effects on apoptosis and cell proliferation and believe this hypothesis warrants further experimental investigation.

## 1. Introduction

Inflammatory bowel disease (IBD) is a group of intestinal disorders, subdivided into Crohn's disease (CD), ulcerative colitis (UC), and, in the absence of a confident diagnosis, unclassified colitis (IBD-U) [1, 2]. Susceptibility to IBD is to a large extent determined by genetic predisposition [3]. Consistent with that, genetic association studies have uncovered a large number of susceptibility loci in relation to IBD. Recently, a total of 163 IBD loci were identified in a genome-wide analysis, and 110 of those susceptibility loci were shared between CD and UC [4]. Within each susceptibility locus, candidate risk genes have been prioritized, based largely on bioinformatic evaluations of the relationships among genes, the presence of coding single nucleotide polymorphisms (SNPs), or the investigation of gene expression-genotype correlations [4–6].

A linkage disequilibrium block encompassing* IKZF3*,* ZPBP2*,* GSDMB*, and* ORMDL3*, which was previously associated with childhood-onset asthma [7], has been identified as an IBD susceptibility locus [6, 8]. Barrett et al. [6] and McGovern et al. [8] highlighted* ORMDL3* in relation to CD and IBD, respectively, based either on a correlation between genotype and gene expression in lymphoblastoid cell lines [7] or on the biological role and previous implication of* ORMDL3* in diseases involving dysregulated immune responses. More recent studies of UC and CD have emphasized several genes (*GSDMA*,* GSDMB*,* IKZF3*,* ORMDL3*,* PNMT*, and* ZPBP2*) within the same region, using information based on bioinformatics, the presence of coding SNPs, or correlations between genotype and gene expression [4, 5, 9].

**Figure 1:**
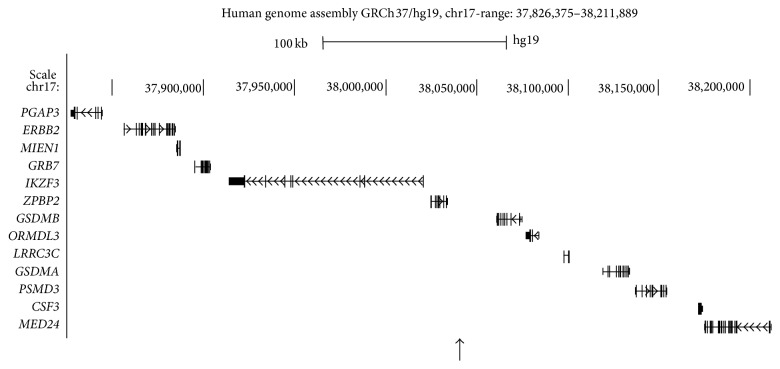
Overview of the genes present within the investigated region. Location of genes in the vicinity of the IBD susceptibility marker rs2872507 (position marked with an upward-pointing arrow) according to the human genome assembly GRCh37/hg19 viewed using the UCSC Genome Browser (http://genome.ucsc.edu/).

The aim of this study was to investigate disease relevant intestinal tissue samples in order to further pinpoint the genes involved in IBD susceptibility. The ileal and colorectal expression of 13 genes within the genetic region of the shared (CD and UC) susceptibility locus rs2872507 (*PGAP3*–*MED24*; Figure 1) was analyzed in relation to genotype, inflammation, and sampling location.

## 2. Material and Methods

### 2.1. Study Samples

Colorectal and ileal mucosal biopsy specimens (*n* = 183) were collected during* routine* endoscopies of 85 adult patients who were being investigated for a known IBD diagnosis or were in the work-up for suspected gastrointestinal disorders (Table 1). Thirty-three patients not afflicted with IBD and without intestinal inflammation were included as noninflamed, non-IBD controls. Study biopsies were collected in parallel to and from the same areas as the biopsies collected for histopathologic assessment. Each biopsy was categorized as “inflamed” or “noninflamed” based on a compound evaluation of endoscopic findings as assessed by one experienced endoscopist (Sven Almer) and on a routine histopathologic assessment for inflammation.

The biopsy specimens used for RNA purification were immersed in RNA*later* RNA stabilization reagent (Qiagen, Hilden, Germany) and stored at 4°C overnight and at −20°C thereafter, awaiting RNA purification.

### 2.2. DNA and RNA Purification

The biopsies were homogenized using TissueRuptor and disposable probes (Qiagen). DNA and RNA were purified using the AllPrep DNA/RNA mini kit (Qiagen) according to the manufacturer's instructions, either manually or using the automated QIAcube system (Qiagen). Concentration and purity were spectrophotometrically measured using a Nanodrop ND-1000 (Thermo Fisher Scientific Inc., Waltham, MA, USA), and RNA integrity was assessed using the RNA integrity number with a 2100 Bioanalyzer (Agilent technologies, Santa Clara, CA). RNasin plus RNase inhibitor (Promega Corporation, Madison, WI, USA) was added to the RNA.

### 2.3. Reverse Transcription

Two preparations of 2 *μ*g RNA from each biopsy were reverse-transcribed in a total volume of 20 *μ*L each using the high capacity cDNA reverse transcription kit with RNase inhibitor (Life Technologies, Carlsbad, CA, USA) according to the manufacturer's instructions. For each biopsy, the resulting cDNA libraries were pooled and stored at −80°C.

### 2.4. Gene Expression Analysis and Data Preprocessing

Assays for gene expression analysis (see S1 Table in Supplementary Material available online at http://dx.doi.org/10.1155/2015/834805) were preloaded onto TaqMan gene expression array cards (Life Technologies). All steps were in accordance with the manufacturer's instructions, using 300 ng sample cDNA per array port (48 different assays).

Threshold cycle (*C*_*T*_) values were established using the ExpressionSuite software version 1.0.1 (Life Technologies). Missing *C*_*T*_ values, caused by low copy numbers, were replaced by the highest *C*_*T*_ value available, increased by one cycle, for the gene in question (GenEx software version 5.4.2, MultiD Analyses, Gothenburg, Sweden). The resulting *C*_*T*_ values were normalized to the average of the selected reference genes (S1 Table) using the GenEx software version 5.4.2. The resulting delta-*C*_*T*_ values were further processed to obtain the relative expression in relation to the sample with the lowest expression of each gene.

### 2.5. Genotyping

The genotype of the SNP susceptibility marker rs2872507 [5, 9] on chromosome 17q12 (http://www.ncbi.nlm.nih.gov/snp) was assessed using 5 ng genomic DNA per sample, a TaqMan SNP genotyping assay (assay ID C_11630970_20), and the TaqMan genotyping master mix (Life Technologies). All genotyping was conducted on the 7500 Fast real-time PCR system using the standard run mode, and the genotypes were generated using the 7500 Fast system SDS software version 2.0.6 (Life Technologies).

### 2.6. Data Analysis

Reference genes were evaluated for low sample-to-sample variation using the GeNorm [10] and NormFinder [11] algorithms in the GenEx software version 5.4.2.

In order to reduce potential confounding effects on the analysis of genotype and gene expression, the samples were stratified based on inflammatory status (inflamed versus noninflamed) and sampling location (ileum versus colon). Multiple colorectal biopsies within inflamed or noninflamed areas from single individuals were treated as biological replicates (see Effects of Sampling Location on Gene Expression in Results). Additionally, the samples were grouped based on disease status (CD, UC, overall IBD, and non-IBD).

The gene expression was investigated in relation to the genotypes using Spearman's rank correlation test. For group comparisons, the Mann-Whitney *U* test or the Kruskal-Wallis ANOVA were used, as appropriate. A Bonferroni-corrected *P* value < 0.00385 was considered significant. Statistical analyses were performed using Statistica version 10.0 (StatSoft Inc., Tulsa, OK, USA).

## 3. Results

Only biopsies with concordant results between the macroscopic appearance at endoscopy and the histopathological findings (i.e., “inflamed” or “noninflamed”) were used for further analyses.

### 3.1. Correlation between Genotype and Gene Expression

**Figure 2:**
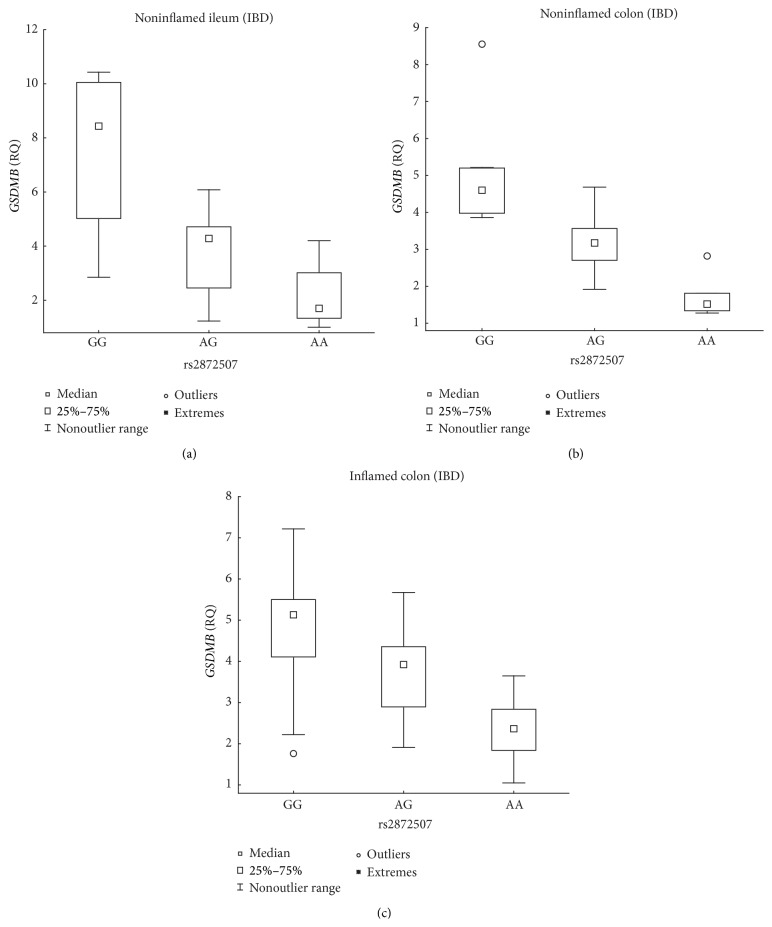
Association between rs2872507 genotype and intestinal* GSDMB* expression in IBD patients. Genotype (A is the IBD risk allele) versus the relative quantity (RQ) of* GSDMB* expression in intestinal biopsies from noninflamed ileal IBD mucosa ((a) *n*_GG_ = 4, *n*_AG_ = 14, *n*_AA_ = 6; *R*_*S*_ = −0.61, *P* = 1.54 × 10^−3^), noninflamed colonic IBD mucosa ((b) *n*_GG_ = 8, *n*_AG_ = 14, *n*_AA_ = 7; *R*_*S*_ = −0.87, *P* = 7.18 × 10^−10^), and inflamed colonic IBD mucosa ((c) *n*_GG_ = 9, *n*_AG_ = 12, *n*_AA_ = 7; *R*_*S*_ = −0.53, *P* = 4.12 × 10^−3^).

**Figure 3:**
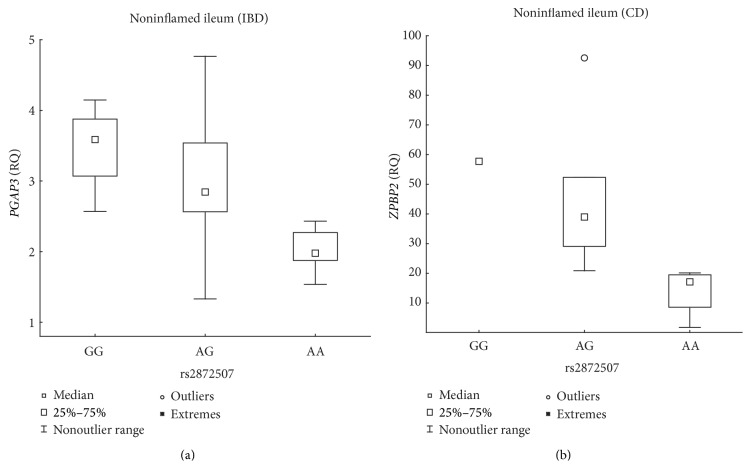
Association between rs2872507 genotype and ileal* PGAP3* expression in IBD patients. Genotype (A is the IBD risk allele) versus the relative quantity (RQ) of* PGAP3* expression in intestinal biopsies from noninflamed ileal IBD mucosa ((a) *n*_GG_ = 4, *n*_AG_ = 14, *n*_AA_ = 6; *R*_*S*_ = −0.60, *P* = 2.00 × 10^−3^) or the RQ of* ZPBP2* expression in noninflamed ileal CD mucosa ((b) *n*_GG_ = 1, *n*_AG_ = 6, *n*_AA_ = 4; *R*_*S*_ = −0.86, *P* = 7.68 × 10^−4^).

The susceptibility allele (A) of the rs2872507 locus (Figure 1) was associated with the reduced expression of* Gasdermin B* (*GSDMB*) in intestinal biopsies from patients with IBD; the associations for noninflamed ileum and noninflamed colon were significant and that for inflamed colon was borderline significant (Table 2, Figure 2). The negative correlation between the number of susceptibility alleles and* GSDMB* expression was also noted separately for CD and UC, although the correlation only reached nominal significance (*P* < 0.05) in noninflamed ileal biopsies and inflamed colonic biopsies from patients with UC (Table 2). Among the patients with IBD, the expression of* GSDMB* was at least 2.17-fold lower in individuals homozygous for the susceptibility allele compared with that in individuals homozygous for the opposite allele (Figure 2). The susceptibility allele was further associated with the reduced expression of* PGAP3* in noninflamed ileum biopsies from the overall IBD group and with the reduced expression of* ZPBP2* in noninflamed ileum biopsies from the CD group (Table 2, Figure 3), with the expression of* PGAP3* reduced 1.81-fold (2 versus 0 susceptibility alleles) and that of* ZPBP2* reduced 2.27-fold (1 versus 0 susceptibility alleles). A correlation between the number of susceptibility alleles and* PGAP3* expression was also identified with nominal significance (*P* < 0.05) separately for CD and UC.

**Figure 4:**
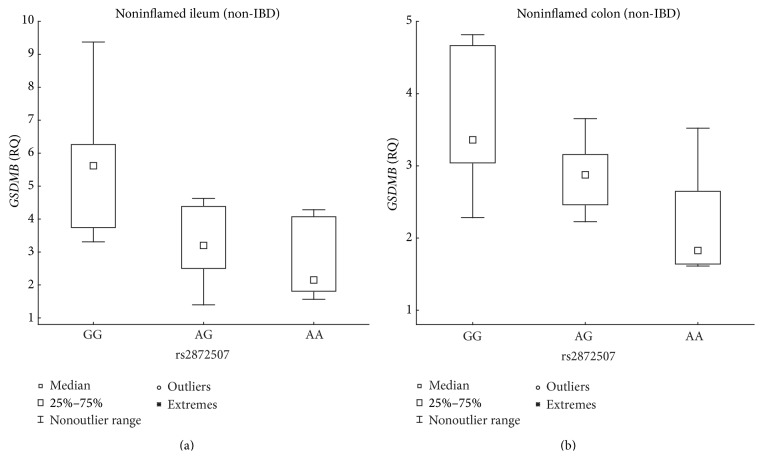
Association between rs2872507 genotype and intestinal* GSDMB* expression in non-IBD patients. Genotype (A is the IBD risk allele) versus the relative quantity (RQ) of* GSDMB* expression in intestinal biopsies from noninflamed ileal non-IBD mucosa ((a) *n*_GG_ = 6, *n*_AG_ = 11, *n*_AA_ = 7; *R*_*S*_ = −0.59, *P* = 2.38 × 10^−3^) or noninflamed colonic non-IBD mucosa ((b) *n*_GG_ = 10, *n*_AG_ = 15, *n*_AA_ = 8; *R*_*S*_ = −0.58, *P* = 3.69 × 10^−4^).

**Figure 5:**
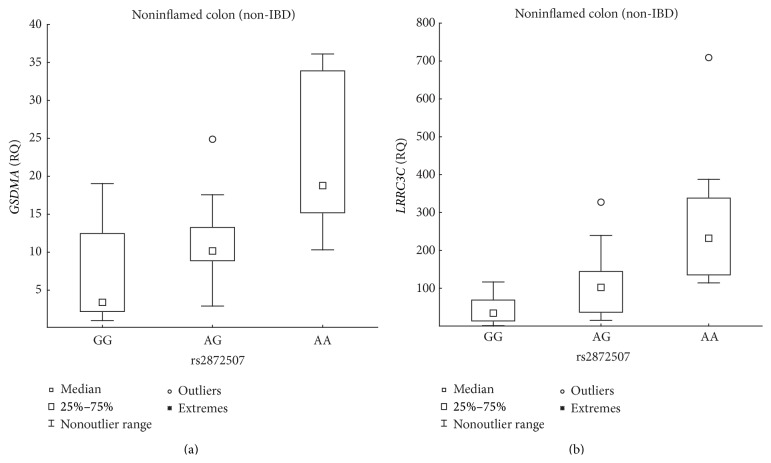
Association between rs2872507 genotype and colonic expression of* GSDMA* and* LRRC3C* in non-IBD patients. Genotype (A is the IBD risk allele) versus the relative quantity (RQ) of* GSDMA* ((a) *n*_GG_ = 10, *n*_AG_ = 15, *n*_AA_ = 8; *R*_*S*_ = 0.61, *P* = 1.61 × 10^−4^) expression or the RQ of* LRRC3C* ((b) *n*_GG_ = 10, *n*_AG_ = 15, *n*_AA_ = 8; *R*_*S*_ = 0.69, *P* = 7.79 × 10^−6^) expression in noninflamed colonic biopsies from non-IBD mucosa.

A negative correlation between* GSDMB* expression and the number of susceptibility alleles was also present in the noninflamed, non-IBD ileal and colonic biopsy samples (Table 2, Figure 4), and the expression was lower at least 1.84-fold in individuals homozygous for the IBD susceptibility allele compared with that in individuals homozygous for the opposite allele. Furthermore, the IBD susceptibility allele was associated with the increased expression of* GSDMA* and* LLRC3C* in noninflamed, non-IBD colonic samples (Table 2, Figure 5) and with expression levels increased 5.53-fold and 6.78-fold, respectively, in individuals homozygous for the susceptibility allele compared with those in individuals homozygous for the opposite allele.

### 3.2. Inflammatory Effects on Gene Expression

Five of the genes displayed either modestly decreased (*MIEN1*,* PGAP3*,* GRB7*, and* ERBB2*; 1.24–2.01-fold) or increased expression (*IKZF3*; 2.41-fold) in biopsies from inflamed, compared with noninflamed, colonic IBD mucosa (Table 3). Similar effects were seen separately for CD and UC, although only nominal significance (*P* < 0.05) was reached for* IKZF3* in CD and for* MIEN1* in both UC and CD.* CSF3* expression was increased at least 116-fold in inflamed, compared with noninflamed, biopsies from the overall IBD group and from the separate disease subentities (Table 3).

### 3.3. Drug-Mediated Effects on Gene Expression

Drug-mediated effects on gene expression were investigated to elucidate a potential explanation for the observed differences in the relationships between genotype and gene expression among patients with and without IBD, respectively. Colonic gene expression levels were compared among patients with IBD receiving a particular treatment versus those not receiving that treatment. No drug-mediated effects were observed in relation to treatment with thiopurines, aminosalicylates, or corticosteroids (S2 Table). There were too few individuals on treatment with anti-TNF-*α*-antibodies for a meaningful statistical analysis.

### 3.4. Effects of Sampling Location on Gene Expression

No differences in gene expression were observed among four different segments of colon (ascending, transverse, descending, and sigmoid) from the noninflamed intestinal mucosa of individuals without IBD (S3 Table; caecum and rectum were excluded because of a low number of samples). Four genes were differentially expressed between the ileum and the colon, however, with either lower (*PSMD3* and* LRRC3C*, 1.34- and 3.85-fold, resp.) or higher (*ORMDL3* and* IKZF3*, 1.34-fold and 2.23-fold, resp.) expression in the ileum compared with that in the colon (S4 Table).

## 4. Discussion

A large number of genetic susceptibility loci have been uncovered in relation to IBD, with pervasive sharing of genetic susceptibility loci between the two major disease subtypes, UC and CD [4]. In this study, we searched for an association between the risk allele of the shared (UC and CD) susceptibility locus rs2872507 and the expression of genes in the intestinal mucosal. This locus has previously been associated with autoimmune diseases such as type-1 diabetes [12], rheumatoid arthritis [13], primary biliary cirrhosis [14], and childhood-onset asthma [7, 15] and has been shown to affect the number of white blood cells [16, 17].

We found that an increase in the number of susceptibility alleles was associated with the reduced expression of* GSDMB* in all of the investigated biopsy locations in noninflamed control individuals and in patients with IBD, irrespective of the presence of active inflammation. Additionally, the susceptibility allele was associated with the increased expression of* GSDMA* and* LRRC3C* in noninflamed colon biopsies from individuals without IBD and with the reduced expression of* PGAP3* and* ZPBP2* in noninflamed ileum biopsies from individuals in the overall IBD cohort and the CD cohort, respectively. Concurrent increase and decrease in the expression of neighboring genes in relation to a susceptibility haplotype for childhood asthma were previously shown for the same genomic region, possibly due to differential associations between the CCCTC-binding factor (CTCF) and different haplotypes [18]. CTCF function as an architectural protein that modifies the topology of the genome, which can result in differential regulation of neighboring genes [19]. A recent analysis of IBD susceptibility loci highlighted* ORMDL3* as a candidate gene in the same region based on a correlation between genotype and* ORMDL3* expression in lymphoblastoid cell lines, liver tissue, and adipose tissue [4]. Susceptibility alleles might exert tissue-dependent effects on gene expression, which are possibly modulated by disease type and medical treatment [20, 21]. It is conceivable that the more extensive alteration of gene expression in the colon (*GSDMA*,* GSDMB*, and* LRRC3C*) compared with that in the CD ileum (*ZPBP2*) reflects the fact that colonic inflammation may occur in both CD and UC, whereas ileal engagement is restricted to CD. Recently, our group demonstrated an effect of thiopurine treatment on the expression of genes present in networks involving candidate IBD susceptibility genes [22]. Although it is likely that such therapeutic effects also extend to the actual IBD susceptibility genes, no such effects of ongoing medication were observed in the current study.

*GSDMA* and* GSDMB* belong to the Gasdermin family of genes. The expression of* GSDMA* and* GSDMB* has been associated with differentiated epithelial cells and with regions containing proliferating cells or stem cells, respectively, of the esophagus and the gastric mucosa [23, 24].* GSDMA*, but not* GSDMB*, expression was capable of inhibiting cell growth in a colony formation assay and could induce apoptosis [24]. Additionally,* GSDMA* expression was frequently suppressed in esophageal cancer cell lines and gastric cancer cell lines, whereas* GSDMB* was expressed in all investigated cancer cell lines and also showed evidence of gene amplification and overexpression in some cases of gastric cancer [24].

No functional studies have, to our knowledge, been published regarding* LRRC3C* and the single-pass membrane protein that it encodes: leucine-rich repeat- (LRR-) containing protein 3C (LRRC3C). Within the human genome, LRRC3C was most similar to the protein encoded by* LRRC3B*. Based on experiments using transformed carcinoma cell lines, a role in the suppression of cell proliferation has been suggested for* LRRC3B* [25, 26].

*ZPBP2* encodes a protein paralogue of the zona pellucida binding protein [27] and harbors a missense SNP associated with primary biliary cirrhosis [14].

The colonic mucosa is a complex tissue, and the biopsies represent a heterogeneous collection of cell types. We did not determine the cellular origin of gene expression. However, all the genes that were differentially expressed with respect to inflammation were of potential interest in relation to either the inflammatory response or the turnover of epithelial cells. Inflamed, compared with noninflamed, colonic biopsies showed strongly increased transcript levels of* CSF3* and modestly increased transcript levels of* IKZF3*, whereas the transcript levels of* ERBB2*,* GRB7*,* MIEN1*, and* PGAP3* were modestly decreased. The granulocyte colony-stimulating factor (encoded by* CSF3*) stimulates the production, survival, and activity of neutrophils [28], and the granulocyte colony-stimulating factor produced by nonhematopoietic cells promotes neutrophil trafficking to the synovial tissue during collagen-induced arthritis in a murine model of rheumatoid arthritis [29]. Neutrophil infiltration of the intestinal mucosa is a feature of IBD [30]. The transcription factor zinc-finger protein Aiolos (encoded by* IKZF3*) is important for lymphocyte differentiation and shows higher expression in B cells [31]. The overexpression of* IKZF3* inhibited proliferation and suppressed apoptosis in a pre-B acute lymphoblastic leukemia cell line [32].* Ikzf3* knockout mice developed a phenotype similar to human autoimmune systemic lupus erythematosus [33]. Also,* Pgap3* knockout mice developed autoimmune-like symptoms, possibly relating to impaired clearance of apoptotic cells by peritoneal macrophages [34].* ERBB2* [35, 36],* GRB7* [37, 38], and* MIEN1* [39, 40] have all been associated with various carcinomas, affecting cell proliferation and/or cell migration.

Considering the risk genotype-gene expression relationships uncovered in our study, both a dysregulated intestinal epithelium [41–43] and aberrant T-cell proliferation and apoptosis [44] have been implicated in the pathogenesis of IBD. However, due to the relatively small sample size of our study the identified relationships need to be cautiously interpreted and confirmed in an independent, preferably larger, study cohort. The exact causes of IBD remain unclear, but the etiology likely includes complex interactions that depend on, at least, the intestinal microbiome and host factors such as genetic susceptibility, intestinal barrier function, and mucosal immune response [45, 46]. In this context it is notable that a joint effect of variants within the genetic region of our study and human rhinovirus wheezing illness has been observed on the subsequent risk of developing childhood-onset asthma. [47, 48]. Although the mechanism of action is not understood, it is possible that it is pertinent to IBD as well. Microbiome studies in IBD have, so far, mainly focused on alterations in the bacterial flora. However, recent studies have also described alterations in the viral component of the intestinal microbiome of IBD patients [49, 50].

Based on our results and published findings on* GSDMA*,* GSDMB*,* LRRC3C*, and related proteins, we propose that the rs2812507 locus in part affects IBD susceptibility via effects on apoptosis and cell proliferation and believe this hypothesis warrants further experimental investigation.

## Supplementary Material

Table S1 lists the investigated reference genes and candidate IBD susceptibility genes and their associated gene expression assay identification numbers.
Table S2 shows the results from the statistical test of drug-mediated effects on gene expression.
Table S3 shows the results from the statistical test of differential gene expression in relation to colonic sampling locations.
Table S4 shows the results from the statistical test of differential differential gene expression in colonic versus ileal biopsies from non-inflamed, non-IBD mucosa.

## Figures and Tables

**Table 1:** Clinical characteristics of participants.

	IBD (*n* = 52)	Non-IBD (*n* = 33)
Disease (CD/UC/IBDU)	21/29/2	
Disease (non-IBD)		a
Gender (male/female)	23/29	13/20
Age (years)^b^	40 (18–77)	49 (20–82)
Smoker (yes/previous/no/no data)	6/5/35/6	3/2/23/5
Immune-modulating drugs		
Aminosalicylates	24	0
Thiopurines	17	0
Corticosteroids	17	0
Anti-TNF-*α*-antibodies	6	1
Methotrexate	2	1
Tacrolimus	2	1
None	10	33

^a^Patients were referred for investigation of gastrointestinal symptoms (e.g., diarrhea, fecal blood, or abdominal pain) or for screening for colorectal adenomas with the following findings: diverticulosis (*n* = 4), polyps (*n* = 5), low-grade dysplasia adenomas (*n* = 2), colorectal cancer (*n* = 2), hemorrhoids (*n* = 3), radiation proctitis (*n* = 1), or without pathological findings (*n* = 19). There were two instances of simultaneous diverticulosis and polyps and one instance of simultaneous diverticulosis and hemorrhoids.

^b^Median (range) values are given.

**Table 2:** Spearman's rank correlation between the genotype of rs2872507 (number of susceptibility alleles) and gene expression (dCt-values) in intestinal biopsy samples from different study groups^a^.

Genes^b^	NI_N_il	NI_N_co	IBD_N_il	IBD_N_co	IBD_I_co	CD_N_il	CD_N_co	UC_N_il	UC_N_co	UC_I_co
	*n* = 24	*n* = 33	*n* = 24	*n* = 29	*n* = 28	*n* = 11	*n* = 12	*n* = 12	*n* = 16	*n* = 19
	*P* value	*P* value	*P* value	*P* value	*P* value	*P* value	*P* value	*P* value	*P* value	*P* value
*PGAP3*	0.37	6.8 × 10^−03^	2.0 × 10^−03^	0.019	0.017	0.030	0.43	0.29	0.060	0.59
*ERBB2*	0.64	0.57	0.69	0.27	0.23	0.91	0.31	0.50	0.68	0.89
*MIEN1*	0.66	0.89	0.034	0.15	0.50	0.79	0.35	0.085	0.023	0.27
*GRB7*	0.93	0.71	0.21	0.61	0.99	0.51	0.93	0.31	0.93	0.50
*IKZF3*	0.88	0.85	0.69	0.43	0.47	0.42	0.39	0.80	0.92	0.27
*ZPBP2*	0.24	0.59	0.84	0.33	0.17	7.7 × 10^−04^	0.95	0.76	0.078	0.064
*GSDMB*	2.4 × 10^−03^	3.7 × 10^−04^	1.5 × 10^−03^	7.2 × 10^−10^	4.1 × 10^−03^	3.8 × 10^−03^	6.2 × 10^−06^	0.035	1.9 × 10^−04^	0.045
*ORMDL3*	0.92	0.77	0.037	0.35	0.47	0.016	0.63	0.24	0.045	0.22
*LRRC3C*	0.83	7.8 × 10^−06^	0.92	0.058	0.42	0.79	0.58	0.21	0.24	0.27
*GSDMA*	0.016	1.6 × 10^−04^	0.92	0.28	0.15	0.28	0.50	0.74	0.50	0.86
*PSMD3*	0.87	0.38	0.51	0.014	0.43	0.45	0.057	0.66	0.14	0.32
*CSF3*	0.42	0.45	0.61	0.66	0.81	0.77	0.31	0.25	0.17	0.10
*MED24*	0.39	0.43	0.16	6.9 × 10^−03^	0.61	0.50	0.085	0.76	0.093	0.21

^a^Disease status_Inflammatory status_sampling location; the disease statuses are Crohn's disease (CD), inflammatory bowel disease (IBD), non-IBD (NI), and ulcerative colitis (UC); the inflammatory statuses are inflamed (I) and noninflamed (N); the sampling locations are colon (co) and ileum (il).

^b^Genes have been arranged (top to bottom) in the order in which they are positioned along the chromosome, and the risk locus (rs2872507) is located between *ZPBP2* and *GSDMB* (closer to *ZPBP2*).

**Table 3:** Inflammatory effects on the colonic mucosal expression of genes in the IBD risk locus (rs2872507)^a^.

Genes^b^	IBD_N_co (*n* = 29) versus IBD_I_co (*n* = 28)		UC_N_co (*n* = 16) versus UC_I_co (*n* = 19)		CD_N_co (*n* = 12) versus CD_I_co (*n* = 7)	
	*P* value	Fold change^c^	*P* value	Fold change^c^	*P* value	Fold change^c^
*PGAP3*	3.7 × 10^−09^	−1.98	9.8 × 10^−06^	−1.92	4.5 × 10^−04^	−2.05
*ERBB2*	1.0 × 10^−09^	−2.12	3.9 × 10^−06^	−2.08	6.2 × 10^−04^	−2.11
*MIEN1*	1.0 × 10^−04^	−1.24	0.014	−1.23	0.010	−1.21
*GRB7*	1.7 × 10^−07^	−2.01	2.9 × 10^−04^	−1.93	8.4 × 10^−04^	−2.01
*IKZF3*	2.4 × 10^−07^	2.41	6.6 × 10^−05^	2.21	6.0 × 10^−03^	2.56
*ZPBP2*	0.21	2.05	0.27	1.96	0.58	2.07
*GSDMB*	0.31	1.11	0.33	1.12	0.97	−1.03
*ORMDL3*	0.65	−1.02	0.56	−1.07	0.64	1.01
*LRRC3C*	0.016	−1.82	0.18	−1.52	0.083	−2.32
*GSDMA*	0.48	1.27	0.96	1.10	0.29	1.71
*PSMD3*	0.024	−1.14	0.030	−1.18	0.33	−1.08
*CSF3*	3.5 × 10^−08^	148.53	4.3 × 10^−05^	115.72	2.7 × 10^−03^	127.34
*MED24*	6.5 × 10^−03^	−1.28	9.3 × 10^−03^	−1.33	0.42	−1.19

^a^Disease status_Inflammatory status_sampling location; the disease statuses are Crohn's disease (CD), inflammatory bowel disease (IBD), and ulcerative colitis (UC); the inflammatory statuses are inflamed (I) and noninflamed (N); the sampling locations are colon (co) and ileum (il).

^b^Genes have been arranged (top to bottom) in the order in which they are positioned along the chromosome.

^c^A negative fold change indicates reduced expression in the inflamed tissue compared with that in the noninflamed tissue.

## References

[1] Geboes K., Van Eyken P. (2009). Inflammatory bowel disease unclassified and indeterminate colitis: the role of the pathologist. *Journal of Clinical Pathology*.

[2] Tremaine W. J. (2012). Is indeterminate colitis determinable?. *Current Gastroenterology Reports*.

[3] Russell R. K., Satsangi J. (2004). IBD: a family affair. *Best Practice & Research: Clinical Gastroenterology*.

[4] Jostins L., Ripke S., Weersma R. K. (2012). Host-microbe interactions have shaped the genetic architecture of inflammatory bowel disease. *Nature*.

[5] Anderson C. A., Boucher G., Lees C. W. (2011). Meta-analysis identifies 29 additional ulcerative colitis risk loci, increasing the number of confirmed associations to 47. *Nature Genetics*.

[6] Barrett J. C., Hansoul S., Nicolae D. L. (2008). Genome-wide association defines more than 30 distinct susceptibility loci for Crohn's disease. *Nature Genetics*.

[7] Moffatt M. F., Kabesch M., Liang L. (2007). Genetic variants regulating *ORMDL*3 expression contribute to the risk of childhood asthma. *Nature*.

[8] McGovern D. P. B., Gardet A., Törkvist L. (2010). Genome-wide association identifies multiple ulcerative colitis susceptibility loci. *Nature Genetics*.

[9] Franke A., McGovern D. P., Barrett J. C. (2010). Genome-wide meta-analysis increases to 71 the number of confirmed Crohn's disease susceptibility loci. *Nature Genetics*.

[10] Vandesompele J., De Preter K., Pattyn F. (2002). Accurate normalization of real-time quantitative RT-PCR data by geometric averaging of multiple internal control genes. *Genome Biology*.

[11] Andersen C. L., Jensen J. L., Ørntoft T. F. (2004). Normalization of real-time quantitative reverse transcription-PCR data: a model-based variance estimation approach to identify genes suited for normalization, applied to bladder and colon cancer data sets. *Cancer Research*.

[12] Barrett J. C., Clayton D. G., Concannon P. (2009). Genome-wide association study and meta-analysis find that over 40 loci affect risk of type 1 diabetes. *Nature Genetics*.

[13] Kurreeman F. A. S., Stahl E. A., Okada Y. (2012). Use of a multiethnic approach to identify rheumatoid- arthritis-susceptibility loci, 1p36 and 17q12. *The American Journal of Human Genetics*.

[14] Hirschfield G. M., Liu X., Han Y. (2010). Variants at IRF5-TNPO3, 17q12-21 and MMEL1 are associated with primary biliary cirrhosis. *Nature Genetics*.

[15] Hao K., Bossé Y., Nickle D. C. (2012). Lung eQTLs to help reveal the molecular underpinnings of asthma. *PLoS Genetics*.

[16] Crosslin D. R., McDavid A., Weston N. (2012). Genetic variants associated with the white blood cell count in 13,923 subjects in the eMERGE Network. *Human Genetics*.

[17] Soranzo N., Spector T. D., Mangino M. (2009). A genome-wide meta-analysis identifies 22 loci associated with eight hematological parameters in the HaemGen consortium. *Nature Genetics*.

[18] Verlaan D. J., Berlivet S., Hunninghake G. M. (2009). Allele-specific chromatin remodeling in the ZPBP2/GSDMB/ORMDL3 locus associated with the risk of asthma and autoimmune disease. *The American Journal of Human Genetics*.

[19] Ong C.-T., Corces V. G. (2014). CTCF: an architectural protein bridging genome topology and function. *Nature Reviews Genetics*.

[20] Grundberg E., Small K. S., Hedman Å. K. (2012). Mapping *cis*- and *trans*-regulatory effects across multiple tissues in twins. *Nature Genetics*.

[21] Kabakchiev B., Silverberg M. S. (2013). Expression quantitative trait loci analysis identifies associations between genotype and gene expression in human intestine. *Gastroenterology*.

[22] Haglund S., Almer S., Peterson C., Söderman J. (2013). Gene expression and thiopurine metabolite profiling in inflammatory bowel disease—novel clues to drug targets and disease mechanisms?. *PLoS ONE*.

[23] Saeki N., Kim D. H., Usui T. (2007). GASDERMIN, suppressed frequently in gastric cancer, is a target of LMO1 in TGF-beta-dependent apoptotic signalling. *Oncogene*.

[24] Saeki N., Usui T., Aoyagi K. (2009). Distinctive expression and function of four GSDM family genes (GSDMA-D) in normal and malignant upper gastrointestinal epithelium. *Genes Chromosomes & Cancer*.

[25] Haraldson K., Kashuba V. I., Dmitriev A. A. (2012). LRRC3B gene is frequently epigenetically inactivated in several epithelial malignancies and inhibits cell growth and replication. *Biochimie*.

[26] Kim M., Kim J.-H., Jang H.-R. (2008). LRRC3B, encoding a leucine-rich repeat-containing protein, is a putative tumor suppressor gene in gastric cancer. *Cancer Research*.

[27] Lin Y.-N., Roy A., Yan W., Burns K. H., Matzuk M. M. (2007). Loss of zona pellucida binding proteins in the acrosomal matrix disrupts acrosome biogenesis and sperm morphogenesis. *Molecular and Cellular Biology*.

[28] Eyles J. L., Roberts A. W., Metcalf D., Wicks I. P. (2006). Granulocyte colony-stimulating factor and neutrophils—forgotten mediators of inflammatory disease. *Nature Clinical Practice Rheumatology*.

[29] Eyles J. L., Hickey M. J., Norman M. U. (2008). A key role for G-CSF induced neutrophil production and trafficking during inflammatory arthritis. *Blood*.

[30] Fournier B. M., Parkos C. A. (2012). The role of neutrophils during intestinal inflammation. *Mucosal Immunology*.

[31] Billot K., Parizot C., Arrouss I. (2010). Differential aiolos expression in human hematopoietic subpopulations. *Leukemia Research*.

[32] Zhuang Y., Li D., Fu J., Shi Q., Lu Y., Ju X. (2014). Overexpression of AIOLOS inhibits cell proliferation and suppresses apoptosis in Nalm-6 cells. *Oncology Reports*.

[33] Sun J., Matthias G., Mihatsch M. J., Georgopoulos K., Matthias P. (2003). Lack of the transcriptional coactivator OBF-1 prevents the development of systemic lupus erythematosus-like phenotypes in Aiolos mutant mice. *Journal of Immunology*.

[34] Wang Y., Murakami Y., Yasui T. (2013). Significance of glycosylphosphatidylinositol-anchored protein enrichment in lipid rafts for the control of autoimmunity. *The Journal of Biological Chemistry*.

[35] Li Q., Wang D., Li J., Chen P. (2011). Clinicopathological and prognostic significance of HER-2/neu and VEGF expression in colon carcinomas. *BMC Cancer*.

[36] Wang S. E. (2011). The functional crosstalk between HER2 tyrosine kinase and TGF-*β* signaling in breast cancer malignancy. *Journal of Signal Transduction*.

[37] Chu P.-Y., Huang L.-Y., Hsu C.-H. (2009). Tyrosine phosphorylation of growth factor receptor-bound protein-7 by focal adhesion kinase in the regulation of cell migration, proliferation, and tumorigenesis. *The Journal of Biological Chemistry*.

[38] Ramsey B., Bai T., Hanlon Newell A. (2011). GRB7 protein over-expression and clinical outcome in breast cancer. *Breast Cancer Research and Treatment*.

[39] Dasgupta S., Wasson L. M., Rauniyar N., Prokai L., Borejdo J., Vishwanatha J. K. (2009). Novel gene C17orf37 in 17q12 amplicon promotes migration and invasion of prostate cancer cells. *Oncogene*.

[40] Evans E. E., Henn A. D., Jonason A. (2006). C35 (C17orf37) is a novel tumor biomarker abundantly expressed in breast cancer. *Molecular Cancer Therapeutics*.

[41] Goto Y., Kiyono H. (2012). Epithelial barrier: an interface for the cross-communication between gut flora and immune system. *Immunological Reviews*.

[42] Uhlig H. H. (2013). Monogenic diseases associated with intestinal inflammation: implications for the understanding of inflammatory bowel disease. *Gut*.

[43] Vereecke L., Beyaert R., van Loo G. (2011). Enterocyte death and intestinal barrier maintenance in homeostasis and disease. *Trends in Molecular Medicine*.

[44] Sturm A., de Souza H. S. P., Fiocchi C. (2008). Mucosal T cell proliferation and apoptosis in inflammatory bowel disease. *Current Drug Targets*.

[45] Knights D., Lassen K. G., Xavier R. J. (2013). Advances in inflammatory bowel disease pathogenesis: linking host genetics and the microbiome. *Gut*.

[46] Wlodarska M., Kostic A., Xavier R. (2015). An integrative view of microbiome-host interactions in inflammatory bowel diseases. *Cell Host & Microbe*.

[47] Caliskan M., Bochkov Y. A., Kreiner-Moller E. (2013). Rhinovirus wheezing illness and genetic risk of childhood-onset asthma. *The New England Journal of Medicine*.

[48] Smit L. A. M., Bouzigon E., Pin I. (2010). 17q21 Variants modify the association between early respiratory infections and asthma. *The European Respiratory Journal*.

[49] Norman J. M., Handley S. A., Baldridge M. T. (2015). Disease-specific alterations in the enteric virome in inflammatory bowel disease. *Cell*.

[50] Wang W., Jovel J., Halloran B. (2015). Metagenomic analysis of microbiome in colon tissue from subjects with inflammatory bowel diseases reveals interplay of viruses and bacteria. *Inflammatory Bowel Diseases*.

