# Supplementary material for: Gene Expression-Genotype Analysis Implicates GSDMA, GSDMB, and LRRC3C as Contributors to Inflammatory Bowel Disease Susceptibility
Source: Biomed Res Int. 2015 Sep 21;2015:834805. doi: 10.1155/2015/834805 (PMC4592899; doi:10.1155/2015/834805)
Supplement: Supplementary file 1 — Table S1 lists the investigated reference genes and candidate IBD susceptibility genes and their associated gene expression assay identification numbers. Table S2 shows the results from the statistical test of drug-mediated effects on gene expression. Table S3 shows the results from the statistical test of differential gene expression in relation to colonic sampling locations. Table S4 shows the results from the statistical test of differential differential gene expression in colonic versus ileal biopsies from non-inflamed, non-IBD mucosa. [file 834805.f1.pdf]

S1 Table. Gene expression assays.

	Gene	Assay ID[a]
**Reference genes**	*CASC3*	Hs00201226_m1
	POP4	Hs00198357_m1
	UBA52	Hs03004332_g1
**IBD candidate genes**	*CSF3*	Hs00357085_g1
	ERBB2	Hs01001580_m1
	GRB7	Hs00918009_g1
	GSDMA	Hs00937853_m1
	GSDMB	Hs00218565_m1
	IKZF3	Hs00232635_m1
	LRRC3C	custom-made assay
	MED24	Hs00207863_m1
	MIEN1	Hs00260553_m1
	ORMDL3	Hs00918021_m1
	PGAP3	Hs00544681_m1
	PSMD3	Hs00160646_m1
	ZPBP2	Hs00418432_m1

[a] Assay identification number according to Life Technologies, Carlsbad, CA, USA. Primer and probe sequences of the custom-made assay for *LRRC3C* are available upon request.

S2 Table. Drug-mediated effects on gene expression in colonic IBD biopsy samples.

	**Non-inflamed IBD colon**			**Inflamed IBD colon**		
	TP *vs.* no TP[a]	AS *vs.* no AS[b]	CS *vs.* no CS[c]	TP *vs.* no TP[a]	AS *vs.* no AS[b]	CS *vs.* no CS[c]
	(7 *vs.* 22)	(14 *vs.* 15)	(8 *vs.* 21)	(9 *vs.* 19)	(15 *vs.* 13)	(12 *vs.* 16)
**Genes**[d]	*P*-value	*P*-value	*P*-value	*P*-value	*P*-value	*P*-value
PGAP3	0.78	0.45	0.40	0.16	0.47	0.13
ERBB2	0.67	0.53	0.10	0.29	0.029	0.13
MIEN1	0.33	0.75	0.49	0.92	0.62	0.40
GRB7	0.90	0.65	0.35	0.22	0.27	0.80
IKZF3	0.71	0.026	0.047	0.96	1.00	0.28
ZPBP2	0.50	0.093	0.021	0.89	0.89	0.98
GSDMB	0.20	0.65	0.79	1.00	0.052	0.16
ORMDL3	0.088	0.40	0.40	0.50	0.75	0.95
LRRC3C	0.26	0.81	0.46	0.56	0.72	0.090
GSDMA	0.50	0.81	0.98	0.31	0.59	0.10
PSMD3	0.53	0.68	0.76	0.41	0.16	0.21
CSF3	0.98	0.95	0.40	0.85	0.79	0.70
MED24	0.86	0.38	0.28	1.00	0.79	0.13

[a] Thiopurine (TP) treatment *vs.* no TP treatment.

[b] Aminosalicylate (AS) treatment *vs.* no AS treatment.

[c] Corticosteroid (CS) treatment *vs.* no CS treatment.

[d] Genes have been arranged (top to bottom) in the order in which they are positioned along the chromosome, and the risk locus (rs2872507) is located between *ZPBP2* and *GSDMB* (closer to *ZPBP2*).

S3 Table. Differential gene expression in relation to colonic sampling locations.

**Genes**[a]	***P*-value**[b]	**Fold change**[c]
PGAP3	0.21	1.21
ERBB2	6.7×10^{-03}	1.36
MIEN1	0.060	1.22
GRB7	0.32	1.14
IKZF3	0.46	1.29
ZPBP2	0.92	1.30
GSDMB	0.050	1.45
ORMDL3	0.27	1.16
LRRC3C	0.41	1.81
GSDMA	0.47	1.94
PSMD3	0.11	1.16
CSF3	0.56	1.42
MED24	0.31	1.43

[a] Genes have been arranged (top to bottom) in the order in which they are positioned along the chromosome.

[b] Biopsy samples from ascending colon (n = 13), transverse colon (n = 11), descending colon (n = 10), and sigmoid colon (n = 23) from the non-inflamed intestinal mucosa of individuals without IBD. Caecum and rectum were excluded because of a low number of samples.

[c] Fold change for the colonic segment with highest expression compared to the colonic segment with the lowest expression.

S4 Table. Differential gene expression in colonic *vs.* ileal biopsies from non-inflamed, non-IBD mucosa.

Genes[a]	*P*-value	Fold change[b]
PGAP3	0.053	-1.21
ERBB2	0.15	1.08
MIEN1	7.1×10^{-03}	-1.21
GRB7	0.69	-1.03
IKZF3	1.7×10^{-04}	2.23
ZPBP2	0.013	2.56
GSDMB	0.16	1.18
ORMDL3	1.8×10^{-05}	1.34
LRRC3C	2.2×10^{-04}	-3.85
GSDMA	0.056	-1.31
PSMD3	6.2×10^{-07}	-1.34
CSF3	0.97	1.39
MED24	0.63	1.03

[a] Genes have been arranged (top to bottom) in the order in which they are positioned along the chromosome.

[b] A negative fold change indicates reduced expression in the ileum compared with that in the colon.
